# Objective and subjective evaluation of trifocal diffractive intraocular Lens after cataract extraction with phacoemulsification: a prospective clinical study

**DOI:** 10.1186/s12886-021-01937-z

**Published:** 2021-04-14

**Authors:** Ahmed A. Zein El-Dein, Ahmed Elmassry, Hazem M. El-Hennawi, Ehab F. Mossallam

**Affiliations:** Ophthalmology Department, Faculty of Medicine, Alexandria University, Champollion Street, Al Attarin, Alexandria, Egypt

**Keywords:** Intraocular lens, Visual acuity, Clinical study

## Abstract

**Background:**

This study aimed to assess visual outcomes, quality of vision and patients’ satisfaction of a trifocal diffractive intraocular lens after cataract surgery with phacoemulsification.

**Results:**

The study included 36 eyes that underwent implantation of trifocal diffractive intraocular lens (IOL). The residual mean postoperative spherical equivalent was − 0.40 ± 0.29 diopters. Mean Uncorrected distance visual acuity was 0.80 ± 0.16 decimal (snellen equivalent 25 ft) while mean Uncorrected intermediate visual acuity was 0.82 ± 0.31 decimal (snellen equivalent 25 ft) and mean Uncorrected near visual acuity (UCNVA) was 0.87 ± 0.20 decimal (snellen equivalent 23 ft). In defocus curve, there was infinitesimal gradual change between the three foci. Contrast sensitivity was just below the inferior limit of normal.

**Conclusion:**

Trifocal diffractive IOL created a true intermediate focus proved by VA and defocus curve and better quality of vision assessed by contrast sensitivity and high order aberration. Moreover, it was safe and effective for correcting distance and near vision in these patients. Most of the patients were very satisfied and achieved spectacle independence.

**Trial registration:**

Registration number and date: NCT04465279 on July 10, 2020.

## Introduction

Cataract represents 33% of all incidences of visual impairment worldwide and is among the main causes of blindness globally [1]. Among the available and valid options, capsular bag implantation of an intraocular lens (IOL) following Phacoemulsification of the crystalline lens, stands as the current standard line of care for patients with cataracts [2, 3]. Monofocal IOLs provide effective distance vision and currently account for the majority of IOLs implantations [2]. However, Patients undergoing cataract surgery with implantation of monofocal IOL may need spectacles to be able to do tasks of near-distance as reading or intermediate-distance as using a computer based on their visual demands [2, 4].

Multifocal IOLs can maintain distance focus and improve near vision have been developed to decrease spectacle dependence [4]. Compared to monofocal IOLs, multifocal IOLs can improve patients’ performance for near-vision tasks; such as reading crafts, social activities and hobbies to a greater extent. Nevertheless, halos in addition to reduced contrast sensitivity have been reported with multifocal IOLs and are known to be the common reasons of patients’ dissatisfaction [5, 6].

Trifocal technology has been developed to create intermediate focus to overcome these difficulties. Continuous reports of the visual outcomes of the trifocal IOLs are encouraging but the quality of vision is still debatable [7–9]. Therefore, this study aims to determine visual outcomes, spectacle independence and patients’ satisfaction of a trifocal diffractive intraocular lens after cataract surgery with phacoemulsification.

## Patients and methods

This was a prospective, non-comparative, non-randomized study. We obtained an informed consent from the patients after explaining the treatment options, the risks and benefits of each procedure, and approval of the study by the ethics committee of Alexandria Faculty of Medicine. The tenets of the Declaration of Helsinki were followed and the trial was registered on clinical trials.gov (NCT04465279 on July 10, 2020).

The study included 36 eyes undergoing implanted trifocal diffractive IOL (Fine-vision, PhysIOL Liège, Belgium) at Ophthalmology department of Faculty of Medicine, Alexandria University, Alexandria, Egypt. We included patient with cataract and no other pathology. Additionally, patients’ desire for spectacle independence after surgery and with realistic expectation. Any patients with ocular comorbidity that affect the end results of the surgery such as history of ocular trauma, irregular corneal astigmatism, pupil abnormalities and capsular or zonular abnormalities that may affect postoperative centration or tilt of the lens (e.g. pseudo exfoliation syndrome and Marfan’s syndrome) were excluded. Refractive astigmatism more than 1.25 D was also excluded.

Preoperatively, all patients had a full ophthalmological examination including refractive status, Goldman applanation tonometry, slit lamp examination, fundus evaluation and biometry with LENSTAR (Haag-Streit)® by using Barrett universal II formula. All cataract surgeries used a standardized sutureless technique, capsulorhexis, hydrodissection, phacoemulsification, irrigation aspiration of cortical remnants, IOL implantation in the capsular bag and hydration of side ports. The used trifocal IOL had a foldable single-piece fully diffractive pupil dependent aspheric IOL. It is made of hydrophilic acrylic with an ultraviolet and blue light inhibitor. It has an optic diameter of 6.15 mm and an overall diameter of 10.75 mm; it has + 3.5D additional power for near vision and + 1.75 D additional power for intermediate vision, consisting of 26 diffractive steps.

### Post-operative assessment

Patients were evaluated 3 months after surgery for refraction, Visual Acuity (VA); using Snellen’s chart for far, Sloan’s chart for intermediate, and Landolt ring chart for near vision. Defocus curve was used to examine monocular and binocular after corrected distant VA refractive error then inserting defocus lenses 0.50-D focus steps from (+ 1.50 to − 3.50 D) in the trial frame. We also assessed contrast sensitivity using the CSV-1000 contrast test (Green Ville-Dayton)®, high order aberration using I-trace aberrometry (Tracy Technology) and filled visual satisfaction questionnaire using patients’ satisfaction questionnaire protocol mediated by PhysIOL for each patient. High order aberration using I-trace aberrometry were additionally assessed after 1 year. Visual acuity, defocus curve and contrast sensitivity were all performed in photopic not mesopic condition.

### Statistical method

Data were fed to the computer and analyzed using IBM SPSS software package version 20.0. Qualitative data were described using number and percent. Quantitative data were described using range (minimum and maximum), mean, standard deviation and median. Paired t-test was used for normally distributed quantitative variables to compare pre- and post-operative data. Significance of the obtained results was judged at the 5% level.

## Results

The study included 18 patients (36 eyes). Of them, there were eight males (44.4%) while the 10 were females (55.6%). The mean age was 55.28 ± 13.14 years with a range from 19 to 76 years. We used IOL power with a range from 10.5 to 28 diopter (D).

### Mean postoperative refraction

Mean sphere were − 0.10 ± 0.39 D with a range from − 0.75 to + 0.75 D while the mean postoperative cylinder was − 0.66 ± 0.24 D with a range from − 0.25 to − 1.25 D and postoperative spherical equivalent was − 0.40 ± 0.29 D with a range from 0.0 to − 1.0 D.

### Postoperative visual acuity

Mean uncorrected distance visual acuity (UCDVA) was 0.80 ± 0.16 decimal snellen equivalent (25 ft) for all patients and 76.5% of eyes had ≥0.7 decimal snellen equivalent (≥30 ft). Mean uncorrected intermediate visual acuity (UCIVA) was 0.82 ± 0.31 decimal snellen equivalent (25 ft) for all patients and 85.5% of eyes had ≥0.5 decimal snellen equivalent (≥40 ft) with 2 eyes had 0.5 decimal. Mean uncorrected near visual acuity (UCNVA) was 0.87 ± 0.20 decimal snellen equivalent (23 ft) for all patients and 88.3% of eyes had ≥0.7 decimal snellen equivalent (≥30 ft). Two eyes were missed during follow up. Table 1 summarized the distribution of the studied eyes according to corrected distance visual acuity. Comparison between UCDVA and corrected distance visual acuity according to far 6 m, intermediate 70 cm and near 40 cm the difference was statistically significant (Table 2). Table 3 and Table 4 summarized the distribution of the studied eyes according to binocular UCDVA and corrected distance visual acuity.

**Table 1:** Distribution of the studied eyes according to corrected distance visual acuity (*n* = 34)

Snellen Equivalent		Decimal	Corrected distance visual acuity		
Feet 20/	Meter 6/		Far 6 m	Intermediate 70 cm	Near 40 cm
**≥ 16**	**≥4.7**	**≥1.2**	3 (8.8%)	3 (8.8%)	1 (2.9%)
**≥ 20**	**≥6.0**	**≥1.0**	13 (38.2%)	8 (23.5%)	13 (38.2%)
**≥ 30**	**≥9.0**	**≥0.7**	17 (50%)	9 (26.5%)	10 (29.4%)
**≥ 40**	**≥12.0**	**≥0.5**	1 (2.9%)	9 (26.5%)	7 (20.6%)
**≥ 63**	**≥18.9**	**≥0.3**	0 (0%)	5 (14.7%)	3 (8.8%)
**Min. – Max.**			−0.08 – 0.22	−0.10 – 0.49	−0.10 – 0.49
**GeoMean (LogMar) ± SD.**			0.0 ± 0.07	0.0 ± 0.17	0.0 ± 0.14
**Median**			0.05	0.10	0.10

**Table 2:** Comparison between UCVA and corrected distance visual acuity according to far 6 m, intermediate 70 cm and near 40 cm (Decimal) Snellen Equivalent meter 6/ (*n* = 34)

	UCVA	Corrected distance visual acuity	t	p
**Far 6 m**				
Min. – Max. (Decimal)	0.50–1.0	0.60–1.20	9.50^*^	< 0.001^*^
Min. – Max. Snellen Equivalent (Feet)	**40–20**	**33–16**		
Mean (Decimal) ± SD.	0.80 ± 0.16	0.91 ± 0.15		
Mean Snellen Equivalent (Feet)	**25**	**22**		
Median	0.80	0.90		
**Intermediate 70 cm**				
Min. – Max. (Decimal)	0.32–1.58	0.32–1.26	2.359^*^	0.024^*^
Min. – Max. Snellen Equivalent (Feet)	**63–12.5**	**18.9–16**		
Mean (Decimal) ± SD.	0.82 ± 0.31	0.77 ± 0.27		
Mean Snellen Equivalent (Feet)	**25**	**26**		
Median	0.79	0.79		
**Near 40 cm**				
Min. – Max. (Decimal)	0.32–1.26	0.32–1.26	2.776^*^	0.009^*^
Min. – Max. Snellen Equivalent (Feet)	**63–16**	**63–16**		
Mean (Decimal) ± SD.	0.87 ± 0.20	0.80 ± 0.22		
Mean Snellen Equivalent (Feet)	**23**	**25**		
Median	0.79	0.79		

t: Paired t-test

p: *p* value for comparing between UCVA and BCVA

*: Statistically significant at *p* ≤ 0.05

**Table 3:** Distribution of the studied eyes according to binocular uncorrected visual acuity [UCVA] (*n* = 17)

Snellen Equivalent		Decimal	UCVA		
Feet 20/	Meter 6/		Far 6 m	Intermediate 70 cm	Near 40 cm
**≥ 16**	**≥4.7**	**≥1.2**	1 (5.9%)	1 (5.9%)	0 (0%)
**≥ 20**	**≥6.0**	**≥1.0**	7 (41.2%)	4 (23.5%)	9 (52.9%)
**≥ 30**	**≥9.0**	**≥0.75**	17 (100%)	13 (76.5%)	16 (94.1%)
**≥ 40**	**≥12.0**	**≥0.5**	17 (100%)	17 (100%)	17 (100%)
**≥ 63**	**≥18.9**	**≥0.3**	17 (100%)	17 (100%)	17 (100%)
**Min. – Max.**			−0.08 – 0.15	−0.15 –0.20	−0.06 – 0.20
**GeoMean (LogMar) ± SD.**			0.0 ± 0.06	0.0 ± 0.10	0.0 ± 0.07
**Median**			0.05	0.10	0.02

**Table 4:** Distribution of the studied eyes according to binocular corrected distance visual acuity (*n* = 17)

Snellen Equivalent		Decimal	Corrected distance visual acuity		
Feet 20/	Meter 6/		Far 6 m	Intermediate 70 cm	Near 40 cm
**≥ 16**	**≥4.7**	**≥1.2**	2 (11.8%)	2 (11.8%)	1 (5.9%)
**≥ 20**	**≥6.0**	**≥1.0**	7 (41.2%)	3 (17.6%)	3 (17.6%)
**≥ 30**	**≥9.0**	**≥0.75**	17 (100%)	14 (82.4%)	14 (82.4%)
**≥ 40**	**≥12.0**	**≥0.5**	17 (100%)	17 (100%)	17 (100%)
**≥ 63**	**≥18.9**	**≥0.3**	17 (100%)	17 (100%)	17 (100%)
**Min. – Max.**			−0.08 – 0.10	−0.10 – 0.20	−0.10 – 0.20
**GeoMean (LogMar) ± SD.**			0.0 ± 0.05	0.0 ± 0.09	0.0 ± 0.08
**Median**			0.02	0.10	0.10

### Defocus curve

**Fig. 1:**
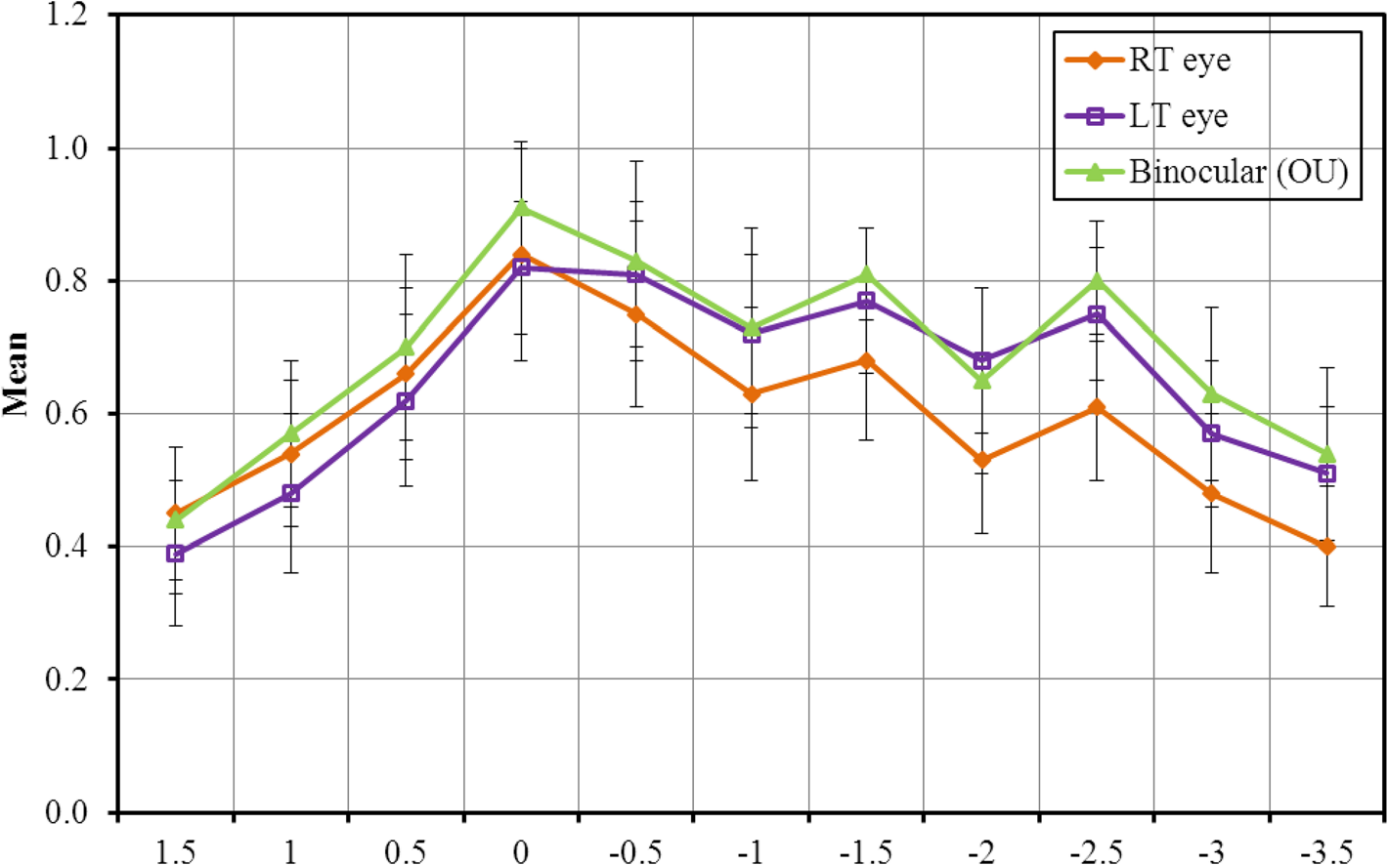
Descriptive analysis of the studied eyes according to defocus curve

Figure 1 shows a descriptive analysis of the studied eyes according to defocus curve. Visual summation occurred when binocular defocus curve was done rather than monocular curve. There was a statistical significance from − 0.5 to − 3.5 between monocular and binocular curves.

### Measurement of high order aberration

A descriptive analysis was done for high order aberration of the studied eyes according to I-Trace. The results after 3 months are summarized in Table 5. It was also done to cases agreed to follow up for a year or more postoperatively and we noticed that there was no significant changes of aberration during this period. Three eyes had PCO were excluded from this analysis. There was no statistical significant difference of I-Trace measurements between 3 months and one year (Tables 6, and 7).

**Table 5:** Descriptive analysis of the studied eyes according to I-Trace

I–Trace	Post operative (3 months)
**HO Total (μ)**	
Min. – Max.	0.06–0.32
Mean ± SD.	0.16 ± 0.08
Median	0.16
**Coma (μ @)**	
Min. – Max.	0.02–0.18
Mean ± SD.	0.07 ± 0.05
Median	0.05
**Spherical (μ)**	
**Positive**	
Min. – Max.	0.0–0.14
Mean ± SD.	0.04 ± 0.04
Median	0.02
**Negative**	
Min. – Max.	− 0.05 – 0.0
Mean ± SD.	− 0.03 ± 0.02
Median	− 0.03
**Trefoil (μ @)**	
Min. – Max.	0.01–0.20
Mean ± SD.	0.08 ± 0.06
Median	0.05
**RMS (mm)**	
Min. – Max.	2.0–3.70
Mean ± SD.	2.73 ± 0.52
Median	2.60

**Table 6:** Comparing the studied cases according to I-Trace after 1 year

I-Trace	Pre-operation	Post operation 1 year	p
**Defocus (μ)**			
**Positive**			
Min. – Max.	0.075–6.288	0.023–1.304	^Z^p=0.109
Mean ± SD.	2.290 ± 1.859	0.266 ± 0.411	
Median	2.233	0.069	
**Negative**			
Min. – Max.	−1.256 – –0.029	−0.437 – –0.008	**–**
Mean ± SD.	− 0.319 ± 0.525	−0.112 ± 0.162	
Median	−0.101	− 0.046	
**Astig (μ @)**			
Min. – Max.	0.014–1.122	0.030–0.619	^Z^p=0.043^*^
Mean ± SD.	0.434 ± 0.372	0.237 ± 0.187	
Median	0.367	0.170	
**HO Total (μ)**			
Min. – Max.	0.066–0.602	0.061–0.500	^Z^p=0.345
Mean ± SD.	0.271 ± 0.163	0.161 ± 0.115	
Median	0.254	0.127	
**Coma (μ @)**			
Min. – Max.	0.013–0.298	0.010–0.200	^Z^p=0.500
Mean ± SD.	0.126 ± 0.087	0.068 ± 0.049	
Median	0.114	0.066	
**Spherical (μ)**			
**Positive**			
Min. – Max.	0.018–0.135	0.0–0.049	**–**
Mean ± SD.	0.056 ± 0.038	0.023 ± 0.020	
Median	0.047	0.021	
**Negative**			
Min. – Max.	−0.472 – –0.003	− 0.169 – –0.001	**–**
Mean ± SD.	− 0.168 ± 0.206	−0.042 ± 0.046	
Median	−0.071	− 0.023	
**2ry Astig (μ @)**			
Min. – Max.	0.005–0.331	0.004–0.149	^Z^p=0.500
Mean ± SD.	0.059 ± 0.081	0.036 ± 0.037	
Median	0.033	0.021	
**Trefoil (μ @)**			
Min. – Max.	0.026–0.242	0.016–0.340	^Z^p=0.893
Mean ± SD.	0.117 ± 0.055	0.104 ± 0.088	
Median	0.129	0.079	
**RMS (mm)**			
Min. – Max.	2.30–5.60	2.0–4.0	^t^p=0.030^*^
Mean ± SD.	3.847 ± 1.053	2.767 ± 0.581	
Median	3.70	2.60	

**t: Paired t-test Z: Wilcoxon signed ranks test**

p: *p* value for comparing between pre-operation and final

*: Statistically significant at *p* ≤ 0.05

**Table 7:** Descriptive analysis of the studied eyes according to I-Trace between 3 months and 1 year

I–Trace	Post operation 3 months	Post operation 1 year	p
**Defocus (μ)**			
**Positive**			
Min. – Max.	0.06–0.53	0.02–0.36	^Z^p = 0.310
Mean ± SD.	0.20 ± 0.16	0.15 ± 0.15	
Median	0.16	0.07	
**Negative**			
Min. – Max.	−0.91 – 0.0	− 0.10 – –0.01	^Z^p = 0.144
Mean ± SD.	− 0.38 ± 0.39	−0.05 ± 0.04	
Median	−0.30	− 0.05	
**Astig (μ @)**			
Min. – Max.	0.05–0.57	0.03–0.62	^Z^p = 0.256
Mean ± SD.	0.21 ± 0.15	0.24 ± 0.19	
Median	0.16	0.17	
**HO Total (μ)**			
Min. – Max.	0.06–0.32	0.06–0.50	^Z^p = 1.000
Mean ± SD.	0.16 ± 0.08	0.16 ± 0.11	
Median	0.16	0.13	
**Coma (μ @)**			
Min. – Max.	0.02–0.18	0.01–0.20	^Z^p = 0.875
Mean ± SD.	0.07 ± 0.05	0.07 ± 0.05	
Median	0.05	0.07	
**Spherical (μ)**			
**Positive**			
Min. – Max.			–
Mean ± SD.	0.12	0.03	
Median			
**Negative**			
Min. – Max.	−0.05 – 0.0	−0.07 – 0.0	^Z^p = 0.893
Mean ± SD.	− 0.03 ± 0.02	−0.03 ± 0.03	
Median	−0.03	− 0.02	
**2ry Astig (μ @)**			
Min. – Max.	0.0–0.12	0.0–0.15	^Z^p = 0.977
Mean ± SD.	0.03 ± 0.03	0.04 ± 0.04	
Median	0.02	0.02	
**Trefoil (μ @)**			
Min. – Max.	0.01–0.20	0.02–0.34	^Z^p = 0.426
Mean ± SD.	0.08 ± 0.06	0.10 ± 0.09	
Median	0.05	0.08	
**RMS (mm)**			
Min. – Max.	2.0–3.70	2.0–4.0	^t^p = 0.832
Mean ± SD.	2.73 ± 0.52	2.77 ± 0.58	
Median	2.60	2.60	

**t: Paired t-test Z: Wilcoxon signed ranks test**

p: *p* value for comparing between pre-operation and final

### Contrast sensitivity

**Fig. 2:**
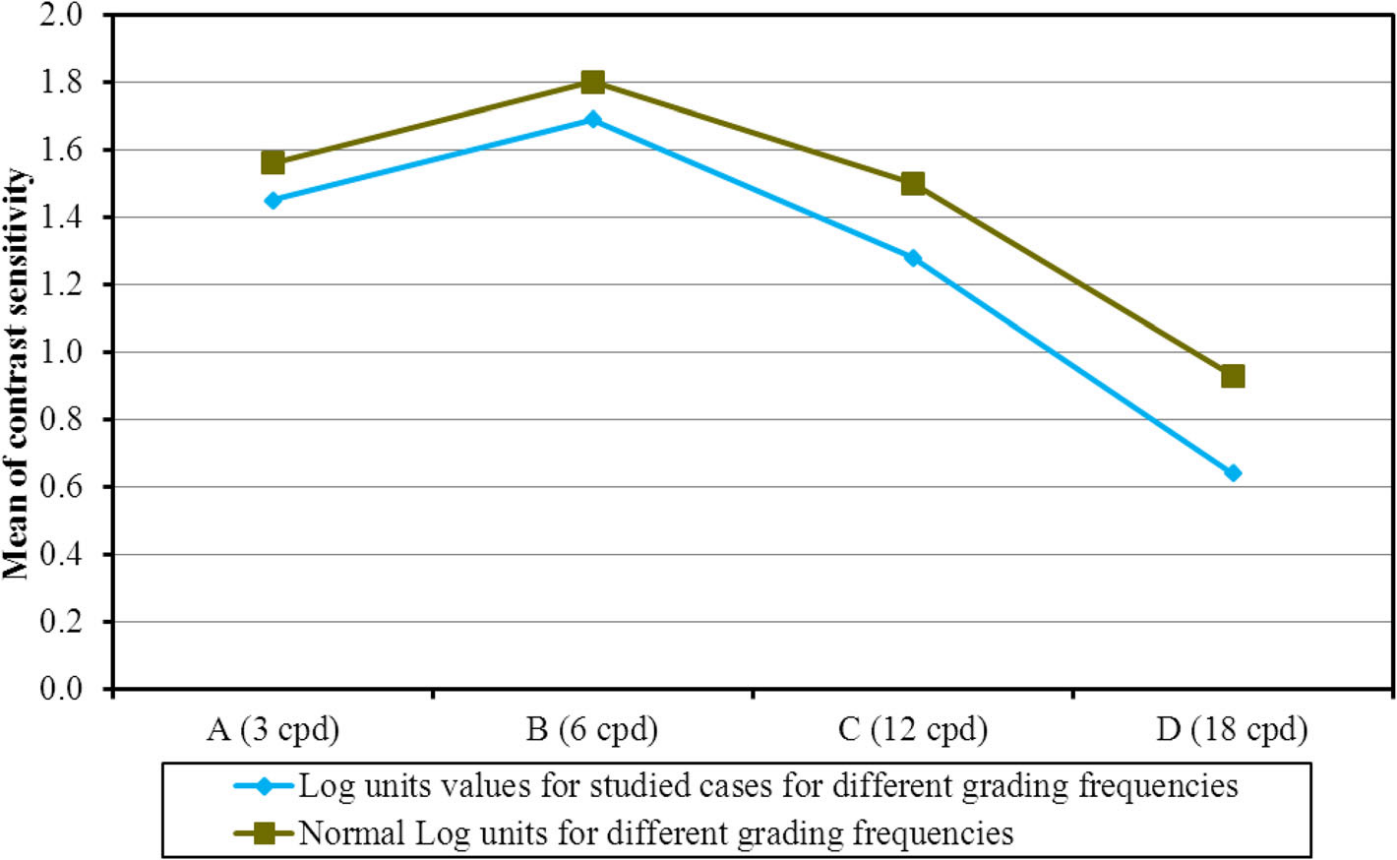
Descriptive analysis of the studied eyes according to contrast sensitivity

There was a mild to moderate decrease in contrast sensitivity values for log units for different spatial frequencies (Fig. 2).

### Patients’ satisfaction questionnaire

One patient complained severe halos that prevent him from driving at night while other two patients mentioned moderate difficulty during driving but they were still capable to drive at night.

## Discussion

As modern technology advances and patients’ expectations increase, the implantation of a multifocal IOL provides an ideal solution for correcting vision to a level that achieves independence from spectacles for distance, intermediate, and near vision [10]. Our study showed that the trifocal IOL in cataract surgery patients is safe and effective at correcting distance, near, and intermediate vision within population. For distance VA, the mean monocular UCDVA of 0.80 ± 0.16 decimal snellen equivalent (25 ft) in our study is similar to Cochener et al. 2018 which had a mean monocular UCDVA of 0.844 ± 0.210 decimal snellen equivalent (23 ft) [11]. However, for intermediate VA, our study revealed that mean UCIVA of 0.82 ± 0.31 decimal snellen equivalent (25 ft) which is superior to the result of Cochener et al. which was 0.57 ± 0.203 decimal SE (36 ft) [11]. This difference may be due to the difference in examination distance which was 70 cm in our study and 60 cm in Cochener et al. It may perform better at 70–80 cm [11]. For near VA, our study showed UCNVA of 0.87 ± 0.20 snellen equivalent (23 ft) which was superior to the mean monocular near UCNVA (0.60 ± 0.13) snellen equivalent (33 ft) of Cochener et al., 2018 [11]. This difference may be due to the difference in postoperative residual refraction in our results and also due to the difference in examination distance which was 40 cm in our study and 30 cm in Cochener et al. [11] Results of a study by Ferreira-Rios et al. showed the mean distance UCDVA was 0.92 ± 0.11 decimal snellen equivalent (22 ft), mean UCIVA at 70 cm 0.91 ± 0.08 decimal snellen equivalent (22 ft) and mean UCNVA 0.90 ± 0.08 decimal snellen equivalent (22 ft) at 40 cm [10]. These result were superior to ours. This difference may be due to patients’ selection and the changes of optical performance in different ages.

Regarding postoperative refraction, Ramon Ruiz-mesa was able to attain slightly less mean residual sphere, cylinder and spherical equivalent for the FineVision group in his study (− 0.08 ± 0.25, 0.14 ± 0.18 and – 0.15 ± 0.25 diopters) compared to our which had slightly more residual refraction (− 0.10 ± 0.39, − 0.66. ± 0.40 and − 0.40 ± 0.29 diopters) [12]. It is worth noting that Ramon Ruiz-mesa used WaveLight AG for optical biometry while we used LENSTAR (Haag-Streit,)®. Moreover, different formulae were used for IOL calculations, Barrett universal II formula was used for all patients in our study [12]. Despite using Barrett universal ll for IOL power calculation we faced post-operative residual errors which may explain VA. Ramon Ruiz-mesa chose Hoffer Q for patients with axial length < 22.0 mm and SRK/T for patients with axial length > 22.0 mm [12]. In our study, the defocus curve shows a gradual change between three foci (far, intermediate, and near) with moderate peak in the near and the intermediate distance. In the study of de Medeiros et al., there was a minimal decrease in VA from distance to intermediate focus (− 1.50 D). In the FineVision group, the defocus curve was continuous from distance to near with a minimal decrease in VA at − 1.50 D (intermediate vision) which was similar to our results [12].

In our study, contrast sensitivity was just below the inferior limit of normal at 3–6 CPD and marked decrease below normal values at 12–18 CPD. In our study, we used CSV 1000. Sheppard et al. reported in their study that the mean values of contrast sensitivity was lower than the normal values and binocular values was significantly higher than monocular values at all spatial frequencies, which were similar with our results [13]. Mean Aberrometry (I-Trace) data for a 4-mm diameter in our study were HO Total (μ) (0.16 ± 0.11), Coma (μ) (0.07 ± 0.05), which were better than Carballo-Alvarez results but spherical aberrations (μ) (0.03 ± 0.02) was similar. Carballo-Alvarez et al. [14], used the aberrometry Topcon KR-1 W after FineVision IOL implantation. Mean outcomes for a mean measured pupil diameter of 4.67 ± 0.67 mm were: High Order RMS 0.41 ± 0.30 μm, coma 0.32 ± 0.22 μm, and Spherical aberrations (SA) 0.21 ± 0.20 μm. The differences may be due to the aberrometry used and pupil size diameter which is 4 mm in our study [14]. Aberrometry after multifocal IOL implantation is not totally reliable [15]. Ocular aberrations are highly pupil-dependent and consequently results obtained with different pupil diameters probably explains the large standard deviation [16]. Cochener et al. found that FineVision IOL had Total HO (μ) less than (0.16 ± 0.09), Coma (μ) (0.10 ± 0.09), Trefoil (μ) (0.08 ± 0.04), and Spherical aberrations (μ) (0.02 ± 0.002) [11]. In our study, we used patients’ satisfaction questionnaire. Overall, most of the patients included in the current study were very satisfied and achieved spectacle independence. Only one patient needed near spectacle for very small characters. Cochener et al. reported that no patients complained of photopic phenomena. In our current study 15.8% complained minimal glare [17]. Glare and halos did not annoy most of our patient during driving at night except one patient. Furthermore, there was no need for lens exchange in any patient and all patients stated that they would be willing to repeat surgery with the same IOL. These results are similar to Cochener et al. [11]

## Conclusion

To recapitulate, trifocal diffractive IOL created a true intermediate focus proved by VA and defocus curve and better quality of vision assessed by contrast sensitivity and high order aberration. Moreover, it was safe and effective for correcting distance and near vision in these patients. Most of the patients were very satisfied and achieved spectacle independence.

## Data Availability

The datasets generated during and analysed during the current study are not available due to privacy/ethical restrictions but are available from the corresponding author on reasonable request.

## Abbreviations

IOL: Intraocular lens
VA: Visual Acuity
UCDVA: Uncorrected distance visual acuity
CNVA: Corrected near visual acuity
UCIVA: Uncorrected intermediate visual acuity
UCNVA: Uncorrected near visual acuity
